# Ba^2+^‐Induced Vasoconstriction as a Model to Investigate the Dynamin Dependence of Biological Processes Regulating Vascular Tone

**DOI:** 10.1002/prp2.70184

**Published:** 2025-10-18

**Authors:** Mariangela Gentile, Alice Panti, Eugenio Paccagnini, Pietro Lupetti, Sergio Bova, Fabio Fusi

**Affiliations:** ^1^ Department of Life Sciences University of Siena Siena Italy; ^2^ Department of Pharmaceutical and Pharmacological Sciences University of Padova Padova Italy; ^3^ Department of Biotechnology, Chemistry and Pharmacy University of Siena Siena Italy

**Keywords:** Ba^2+^‐induced vascular contraction, dynamin, mdivi‐1, mitochondrial fission

## Abstract

Understanding the mechanisms underpinning vascular smooth muscle contraction, which are critical targets for cardiovascular disease treatment, is essential for developing novel therapeutic agents. Recently, the role of mitochondrial fission as a key modulatory event in the vascular contractile process has been questioned. Therefore, the present study, conducted on ex vivo rat aorta rings, aimed to elucidate its role. As mitochondrial dynamics is a Ca^2+^‐dependent process, experiments were performed using preparations incubated in a Ca^2+^‐free medium, depleted of sarcoplasmic reticulum Ca^2+^ content, and stimulated by Ba^2+^. Contractile responses evoked by Ba^2+^, either alone or in the presence of phenylephrine or (S)‐(−)‐Bay K 8644, occurred without mitochondrial fission. Furthermore, hallmarks of mitochondrial fusion were observed in rings stimulated by Ba^2+^ alone. The Drp1 inhibitors mdivi‐1 and dynasore antagonized Ba^2+^‐induced contraction, whereas the dynasore analogue dyngo‐4a and the dynamin stimulator ryngo 1–23 synergized with Ba^2+^‐induced contraction. All tested compounds, except mdivi‐1, induced mitochondrial fission, with particularly pronounced effects observed with dynasore. Similar results were obtained in rings stimulated by Ba^2+^ in the presence of either phenylephrine or (S)‐(−)‐Bay K 8644. In conclusion, these findings indicate that rat aorta contraction can occur independently of mitochondrial fission. Moreover, Ba^2+^, used in place of Ca^2+^ as a vasoconstricting agent, provides a valuable experimental framework for identifying off‐target effects of dynamin modulators.

**Abbreviations**
DRP1: Dynamin Related Protein 1
INF2: inverted formin 2
KHS: modified Krebs–Henseleit solution
ROS: reactive oxygen species

## 1 Introduction

Mitochondrial division (fission) is a pivotal event in the regulation and maintenance of mitochondrial function, quality, and distribution, and it plays a significant role in the modulation of various cellular processes. Moreover, mitochondrial fission is now recognized as a terminal event within a signaling pathway that enables cells to detect and respond to external stimuli [1]. The fission process occurs at contact sites between mitochondria and the endoplasmic reticulum. It is ultimately mediated by the dynamin‐like GTPase, Dynamin Related Protein 1 (DRP1), which functions in conjunction with the endoplasmic reticulum‐localized inverted formin 2 (INF2) [2]. The release of Ca^2+^ from the endoplasmic reticulum is essential for activating both DRP1 [1, 3] and INF2 [3, 4].

Ca^2+^ is a well‐established regulator of vascular smooth muscle mechanics; however, Ca^2+^‐independent mechanisms, such as increased myofilament sensitivity to Ca^2+^, also contribute to this process [5]. In many cases, vasoconstrictors elevate intracellular Ca^2+^ concentrations in vascular smooth muscle cells by facilitating Ca^2+^ entry via Ca_V_ channels, as observed with high K^+^ concentrations or Bay K 8644 [6]. Alternatively, they activate the PLC‐IP_3_ ‐PKC pathway (e.g., phenylephrine, endothelin‐1, and vasopressin), which stimulates both Ca^2+^ influx through receptor‐operated and Ca_V_ channels and Ca^2+^ release from the sarcoplasmic reticulum via IP_3_‐sensitive Ca^2+^ channels [7, 8, 9]. The resulting rise in intracellular Ca^2+^ initiates the contractile process. The Rho‐kinase pathway may also play a role in this mechanism [5].

Ba^2+^ has been widely reported to substitute for Ca^2+^ in activating the contractile process in vascular smooth muscle [10]. At μM concentrations, Ba^2+^ is commonly used as an inhibitor of K_ir_ and K₂_P_ channels, whereas mM concentrations are required to affect K_Ca_ and K_V_ channels [11]. This leads to membrane depolarisation, Ca_V_ channel activation, and subsequent Ca^2+^ influx.

Ba^2+^ has been shown to induce contraction in Ca^2+^‐depleted vascular strips even in the absence of extracellular Ca^2+^ [12], as it enters vascular smooth muscle cells via Ca_V_ channels and directly activates contractile proteins, mimicking the behavior of Ca^2+^ [13]. However, Ba^2+^ does not accumulate in the sarcoplasmic reticulum [14], nor does it stimulate Ca^2+^ release from noradrenaline‐sensitive intracellular Ca^2+^ pools [12, 15]. Like Ca^2+^, Ba^2+^ can enter mitochondria, accumulating in Ca^2+^‐containing granules [16].

**FIGURE 1:**
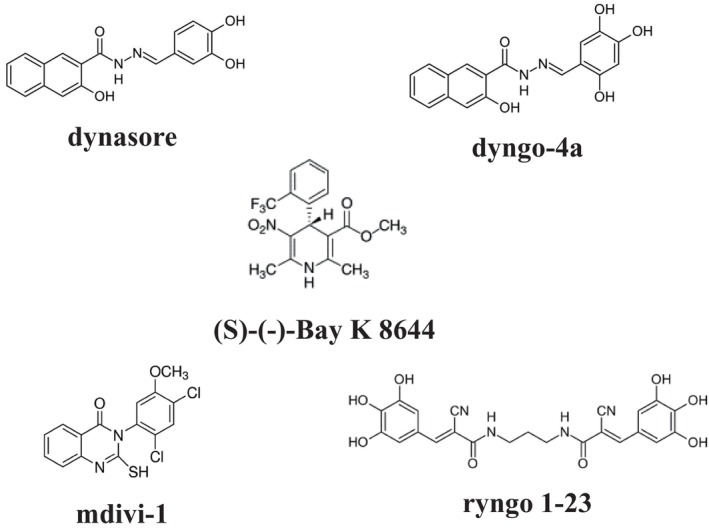
Molecular structures of the compounds used in the study.

Liu et al. [17] and Chen et al. [18] proposed a positive role for mitochondrial fission during vasoconstriction, based on observations that two well‐known DRP1 inhibitors, dynasore and mdivi‐1 (Figure 1), relax both receptor‐ and KCl‐induced contractions. Specifically, they suggested that increased intracellular Ca^2+^ concentrations, or the Ca^2+^‐independent activation of Rho‐associated protein kinase triggered by receptor stimulation (via α₁‐adrenergic or endothelin‐1 receptors) or membrane depolarisation, induces mitochondrial fission. This process purportedly releases reactive oxygen species that enhance vascular contraction.

However, this hypothesis has been challenged by recent multidisciplinary studies demonstrating that dynasore and mdivi‐1 exhibit off‐target actions unrelated to DRP1 inhibition, which fully account for their vasorelaxant effects [19, 20].

As both mitochondrial fission and vasoconstriction are Ca^2+^‐dependent processes, it remains impossible to definitively ascertain the contribution of mitochondrial fission to vasoconstriction in the presence of Ca^2+^, due to the lack of selective DRP1 inhibitors. Consequently, in this study, it has been hypothesized that Ba^2+^ cannot substitute for Ca^2+^ in the activation of DRP1, thereby allowing Ba^2+^‐induced contraction to serve as a model system in which vasoconstriction is decoupled from mitochondrial fission. This approach facilitates a more precise understanding of the relationship between these two phenomena.

The present investigation examined whether mitochondrial fission occurs in rat aortic rings contracted by Ba^2+^. The results indicate that mitochondrial fission did not occur during Ba^2+^‐induced vasoconstriction, suggesting that mitochondrial fission is not an obligatory event in vascular contraction induced by this agent. Furthermore, the observation that both mdivi‐1 and dynasore inhibited Ba^2+^‐induced contractions confirms that these compounds exert vasorelaxant effects on vascular smooth muscle independently of dynamin.

## 2 Materials and Methods

### 2.1 Animal Care Statement

All study procedures adhered strictly to the European Union Guidelines for the Care and Use of Laboratory Animals (European Union Directive 2010/63/EU) and received approval from the Animal Care and Ethics Committee of the University of Siena and the Italian Department of Health (7DF19.N.SJQ on 12 April 2024).

### 2.2 Aorta Ring Preparation

Male Wistar rats (260–360 g), purchased from Charles River Italia (Calco, Italy), were housed in an animal facility with a temperature of 25°C ± 1°C and a 12:12 h dark–light cycle. They were fed a standardized diet and had access to drinking water *ad libitum*. Animals were anesthetized with isoflurane (4%) and O_2_ gas mixture using Fluovac (Harvard Apparatus, Holliston, Massachusetts, USA), before decapitation and exsanguination. The abdominal aorta was rapidly removed, immersed in a modified Krebs–Henseleit solution (KHS; see below for composition), and processed. Rings (2–4 mm wide) were cut, and the endothelium was removed by gently rubbing the lumen of the ring with a forceps tip to avoid interference by endothelium‐derived factors and allow an easier interpretation of the results. Rings were mounted under a passive tension of 1 g in organ baths filled with KHS (composition in mM: 118 NaCl, 4.75 KCl, 1.19 KH_2_PO_4_, 1.19 MgSO_4_, 25 NaHCO_3_, 11.5 glucose, 2.5 CaCl_2_, gassed with carboxygen; pH 7.4) for isometric tension recordings using a digital PowerLab data acquisition system (PowerLab 8/30; ADInstruments, Castle Hill, Australia). After a 60‐min equilibration period, ring viability was accomplished by measuring the response to 0.3 μM phenylephrine (pharmaco‐mechanical coupling) and 60 mM KCl (electro‐mechanical coupling). The absence of a functional endothelium was proved by the lack of response to 10 μM acetylcholine added at the plateau of phenylephrine‐induced contraction [21].

### 2.3 Functional Experiments

Ba^2+^‐induced contraction was investigated under three different experimental conditions: (1) Ba^2+^ alone; (2) Ba^2+^ in rings pre‐treated with phenylephrine; and (3) Ba^2+^ in rings pre‐treated with (S)‐(−)‐Bay K 8644.

#### 2.3.1 Ba^2+^‐Induced Contraction

Rings were washed and incubated in a PO_4_^3−^‐ and Ca^2+^‐free KHS containing 1 mM EGTA, then stimulated by 1 μM phenylephrine to deplete the sarcoplasmic reticulum. This procedure was repeated three times. The success of the depletion protocol was confirmed by the absence of a contractile response to the final application of phenylephrine, which was subsequently washed out with a PO_4_^3−^‐ and Ca^2+^‐free KHS containing 1 mM EGTA. Drugs or vehicle (DMSO) were incubated in the same solution for 25 min, after which 3 mM BaCl_2_ was added to induce muscle contraction. Pre‐incubation was necessary because Ba^2+^‐induced contraction spontaneously declined once reaching its maximum, complicating the interpretation of results if drugs were added after stimulation. The contractile response was expressed as a percentage of the contraction induced by 60 mM KCl during the functional test.

#### 2.3.2 Ba^2+^‐Induced Contraction in the Presence of Phenylephrine

Rings were stimulated by 1 μM phenylephrine and then washed five times (once every 10–15 min) with a PO_4_^3−^‐ and Ca^2+^‐free KHS containing 1 mM EGTA. Under these experimental conditions, 1 μM phenylephrine was added again to confirm sarcoplasmic reticulum depletion. While phenylephrine was present, rings were then stimulated by 3 mM BaCl_2_. Drugs or the vehicle were added at the plateau of Ba^2+^‐induced contraction, as preliminary observations showed that active muscle tone remained stable. The response was expressed as a percentage of the contraction induced by the first stimulation with 1 μM phenylephrine.

#### 2.3.3 Ba^2+^‐Induced Contraction in the Presence of (S)‐(−)‐Bay K 8644

Rings were stimulated by 1 μM phenylephrine and then washed five times (once every 10–15 min) with a PO_4_^3−^‐ and Ca^2+^‐free KHS containing 1 mM EGTA. Under these conditions, (S)‐(−)‐Bay K 8644 (1 μM) was added, followed after 10 min by the vehicle or drug, which was incubated for an additional 10 min. Then, the rings were stimulated by 3 mM BaCl_2_. Drug pre‐incubation was necessary because Ba^2+^‐induced contraction spontaneously declined once reaching its maximum, complicating the interpretation of results if drugs were added after stimulation. The response was expressed as a percentage of the contraction induced by the first stimulation with 1 μM phenylephrine.

At the end of each experimental protocol, nifedipine (1 μM) and/or sodium nitroprusside (100 μM) was added to test the viability of the smooth muscle.

### 2.4 Transmission Electron Microscopy

At the end of each experimental protocol detailed above, rings were unmounted, rinsed four times in 0.1 M sodium cacodylate buffer (pH 7.2), and fixed in 2.5% glutaraldehyde diluted in cacodylate buffer for 3 h at 4°C. Then samples were washed in cacodylate buffer overnight and post‐fixed in 1% osmium tetroxide for 1 h at 4°C. Following an additional rinse in cacodylate buffer, the samples were dehydrated through a graded series of ethanol and embedded in Epon epoxy resin, with the samples positioned to obtain cross‐sections. Aorta ring thin sections (70 nm) were prepared with a Reichert Ultracut E ultramicrotome (Reichert‐Jung AG, Wien, Austria) and collected on 150 mesh copper grids. These sections were routinely stained with uranyl acetate and lead citrate, then observed using an FEI Tecnai G2 Spirit transmission electron microscope (FEI Company, Hillsboro, OR, USA) at an electron accelerating voltage of 120 kV, equipped with a TVIPS TemCam‐F216 CMOS camera (TVIPS GmbH, Gauting, Germany). Images of the samples were acquired at 6800X magnification, and the mitochondria were manually measured using ImageJ software (ver 1.53j, NIH, http://imagej.nih.gov/ij/), considering the following parameters: aspect ratio (major axis/minor axis) and roundness [4 × area/(π × major axis^2^)].

### 2.5 Materials

The chemicals used were: acetylcholine, dyngo‐4a, ryngo 1–23, mdivi‐1, (S)‐(−)‐Bay K 8644, nifedipine, and phenylephrine (Merk Life Science, Milan, Italy); sodium nitroprusside (Riedel‐De Haen AG, Seelze Hannover, Germany); dynasore (Abcam, Milan, Italy); sodium cacodylate (Acros organics Geel, Belgium); glutaraldehyde and osmium tetroxide (Ted Pella Inc., Redding, CA, USA); Epon epoxy resin (Serva Electrophoresis GmbH, Heidelberg, Germany); uranyl acetate (Fluka Chemie AG, Buchs, Switzerland); and lead citrate (Carlo Erba Reagents Srl, Cornaredo, Milan, Italy). Phenylephrine was solubilized in 0.1 M HCl, nifedipine in ethanol, dynasore, ryngo 1–23, mdivi‐1, and dyngo‐4a in DMSO, and sodium nitroprusside in distilled water. Neither DMSO nor ethanol (maximal concentration of 0.1%, v/v) affected vascular responses (data not shown).

### 2.6 Statistical Analysis

LabChart 7.3.7 Pro (PowerLab; ADInstruments, Castle Hill, Australia) and GraphPad Prism 5.04 (GraphPad Software Inc.) performed the analysis of data, which are reported as mean ± SEM; *n* is the number of rings analyzed (indicated in parentheses), isolated from at least three animals. GraphPad Prism 5.04 (GraphPad Software Inc.) performed the statistical analysis by one‐way ANOVA (followed by Dunnett post hoc test) or Student's *t* test for either paired or unpaired samples (two‐tailed). *p* < 0.05 was considered significant.

### 2.7 Nomenclature of Targets and Ligands

Key protein targets and ligands in this article are hyperlinked to corresponding entries in http://www.guidetopharmacology.org, the common portal for data from the IUPHAR/BPS Guide to PHARMACOLOGY [22], and are permanently archived in the Concise Guide to PHARMACOLOGY 2019/20 [23].

## 3 Results

### 3.1 Mechanical Activity and Mitochondrial Dynamics Assessment in Aorta Rings Stimulated by BaCl_2_

**FIGURE 2:**
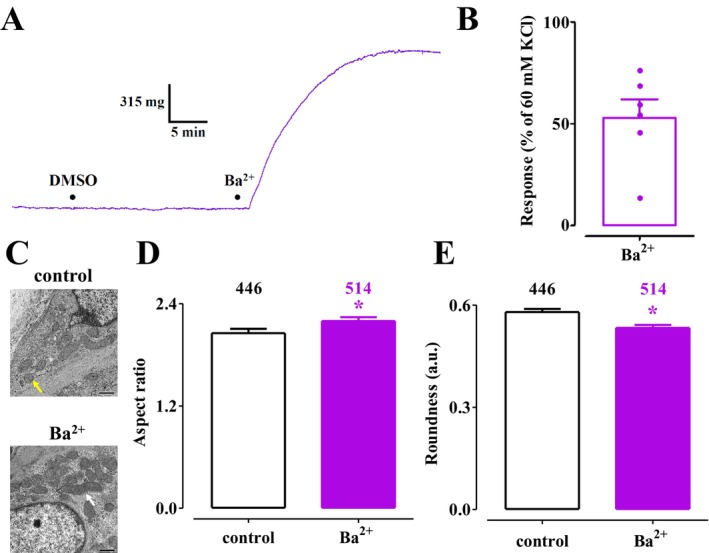
Effect of Ba^2+^ on rat aorta ring muscle tone and mitochondrial dynamics. (A) Trace (representative of six experiments) showing tension developed by a Ca^2+^‐depleted aorta ring maintained in a PO_4_^3−^‐ and Ca^2+^‐free KHS containing 1 mM EGTA, in response to 3 mM BaCl_2_ in the presence of the vehicle (11.4 mM DMSO). (B) Effect of 3 mM BaCl_2_ on ring muscle tone. On the ordinate scale, the response is expressed as a percentage of the tension evoked by 60 mM KCl in the ring viability test. Columns represent the mean ± SEM (*n* = 6). (C) Transmission electron microscope images captured in aorta rings under control conditions (the yellow arrow points at a fragmented mitochondrion) and following contraction induced by 3 mM BaCl_2_ (the white arrow points at an elongated mitochondrion) (scale bar = 500 nm). (D, E) Summary of transmission electron microscope data: (D) aspect ratio and (E) roundness representative of mitochondrial dynamics. Columns represent the mean ± SEM; the number of mitochondria analyzed per group is indicated above the bars. *p < 0.05 vs. control, Student's *t* test for unpaired samples.

A first series of experiments was conducted to determine whether mitochondrial fission occurred in Ca^2+^‐depleted aorta rings contracted by Ba^2+^ in the absence of extracellular Ca^2+^. Figure 2A presents an original recording of the time course of Ba^2+^‐induced contraction, which reached a plateau after approximately 18 min and corresponded to 52.9% ± 22.2% (*n* = 6) of the contraction evoked by 60 mM KCl in KHS (Figure 2B). Morphological analysis performed at the plateau of the contractile response revealed that mitochondria were 10% more elongated compared to those in unstimulated rings (Figure 2C), as evidenced by an increased aspect ratio and decreased mitochondrial roundness values (Figure 2D,E), thus suggesting an increase in mitochondrial fusion.

**FIGURE 3:**
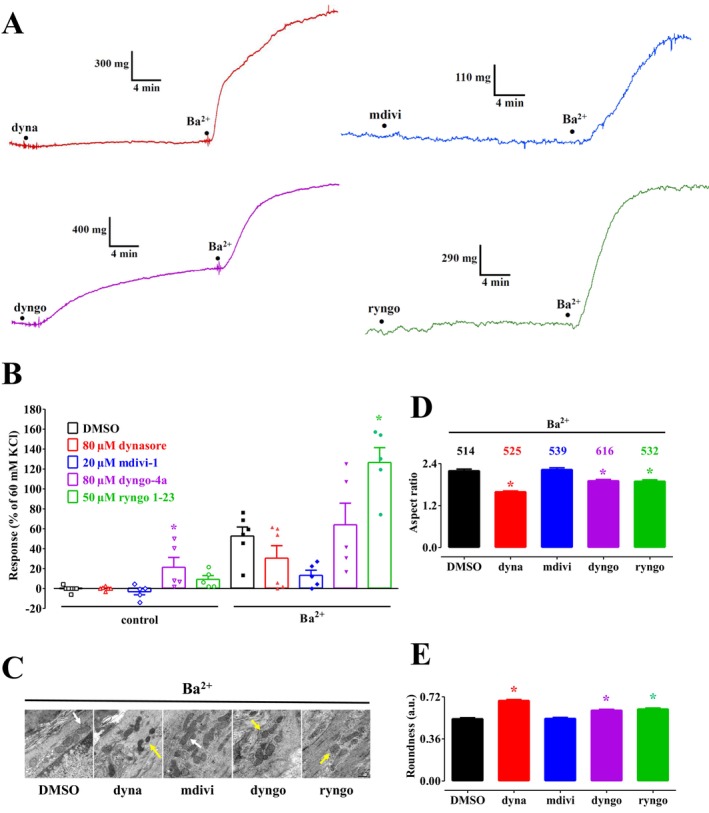
Effect of dynamin modulators on Ba^2+^‐induced contraction and mitochondrial dynamics in rat aorta rings. (A) Traces (representative of five experiments) showing tension developed in response to 3 mM BaCl_2_ in the presence of 80 μM dynasore, 20 μM mdivi‐1, 80 μM dyngo‐4a, or 50 μM ryngo 1–23. (B) Effect of either DMSO, 80 μM dynasore, 20 μM mdivi‐1, 80 μM dyngo‐4a, or 50 μM ryngo 1–23 on ring muscle tone under control conditions (left) and after the addition of 3 mM BaCl_2_ (right). On the ordinate scale, the response is expressed as a percentage of the tension evoked by 60 mM KCl in the ring viability test. Columns represent the mean ± SEM (*n* = 5–6). *p < 0.05 vs. DMSO, Dunnett post hoc test. (C) Transmission electron microscope images of aorta rings contracted by 3 mM BaCl_2_ in the presence of 11.4 mM DMSO, 80 μM dynasore (dyna), 20 μM mdivi‐1 (mdivi), 80 μM dyngo‐4a (dyngo), or 50 μM ryngo 1–23 (ryngo) pre‐incubated for 25 min (scale bar = 500 nm). Yellow arrows point at fragmented mitochondria, whereas white arrows point at elongated mitochondria. (D, E) Summary of transmission electron microscope data: (D) aspect ratio and (E) roundness representative of mitochondrial dynamics. Columns represent the mean ± SEM, with the number of mitochondria analyzed per group shown above the bars. *p < 0.05 vs. DMSO, Dunnett post hoc test.

Under the same experimental conditions, the effects of the DRP1 inhibitors dynasore, dyngo‐4a, and mdivi‐1, as well as the stimulator ryngo 1–23 (Figure 3A), were assessed on Ba^2+^‐induced contraction and mitochondrial dynamics. Figure 3B shows that only dyngo‐4a caused a slight, though significant, contraction during the pre‐incubation period. Ba^2+^‐induced contraction was markedly, though not significantly, inhibited by dynasore (−42%) and mdivi‐1 (−75%), and potentiated by dyngo‐4a (+21%) and ryngo 1–23 (+140%). The effects of DRP1 modulators on mitochondrial aspect ratio and roundness values are summarized in Figure 3C–E. All the compounds tested, except mdivi‐1, caused a significant reduction in mitochondrial aspect ratio and an increase in roundness values, with these effects being particularly pronounced for dynasore, thus suggesting an increase in mitochondrial fission.

### 3.2 Mechanical Activity and Mitochondrial Dynamics Assessment in Phenylephrine Pre‐Treated Aorta Rings Stimulated by BaCl_2_

**FIGURE 4:**
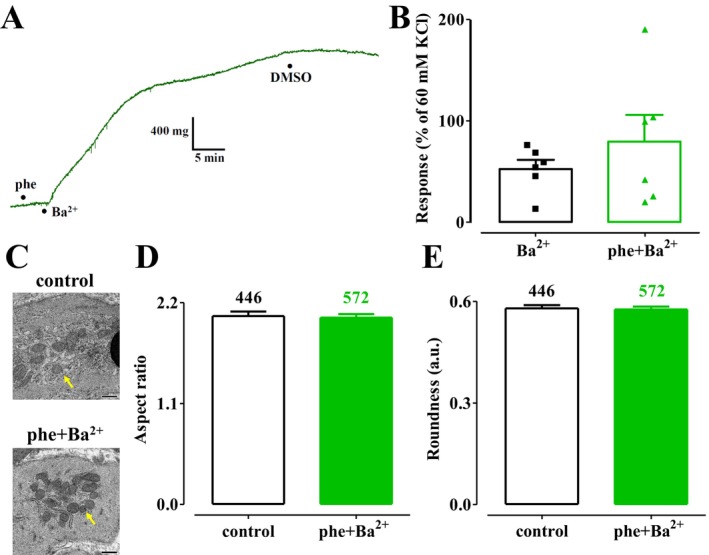
Effect of phenylephrine plus Ba^2+^ on rat aorta ring muscle tone and mitochondrial dynamics. (A) Trace (representative of six experiments) showing the effect of the vehicle (11.4 mM DMSO) added at the plateau of the tension developed in response to 3 mM BaCl_2_ in the presence of 1 μM phenylephrine. (B) Effect of 3 mM BaCl_2_, in the absence (same data as in Figure 2B) or presence of 1 μM phenylephrine, on ring muscle tone. On the ordinate scale, the response is expressed as a percentage of the tension evoked by 60 mM KCl in the ring viability test. Columns represent the mean ± SEM (*n* = 6). (C) Transmission electron microscope images of aorta rings under control conditions (the yellow arrow points at a fragmented mitochondrion) and contracted by 3 mM BaCl_2_ in the presence of 1 μM phenylephrine (the white arrow points at an elongated mitochondrion) (scale bar = 500 nm). (D, E) Summary of transmission electron microscope data: (D) aspect ratio and (E) roundness representative of mitochondrial dynamics. Columns represent the mean ± SEM, with the number of mitochondria analyzed per group shown above the bars.

As phenylephrine increases mitochondrial fission in rat aorta rings bathed in Ca^2+^‐containing KHS [19, 20], a second series of experiments was conducted to evaluate whether the same effect could be reproduced in Ca^2+^‐depleted preparations stimulated by Ba^2+^ in the absence of extracellular Ca^2+^. As illustrated in Figure 4A, 1 μM phenylephrine did not alter ring muscle tone in the absence of Ba^2+^. The subsequent addition of 3 mM Ba^2+^ elicited a stable contraction, reaching a plateau after approximately 40 min and showing a response about 50% greater than that induced by Ba^2+^ alone (Figure 4B). However, this difference was not statistically significant. Mitochondrial aspect ratio and roundness values, measured after the addition of phenylephrine (1.725 ± 0.042 and 0.652 ± 0.011; *n* = 292), were not different from those measured in its absence (1.714 ± 0.047, *p* = 0.8615, and 0.672 ± 0.012, *p* = 0.2254; *n* = 295). The values measured at the plateau of Ba^2+^‐induced contraction, in the presence of phenylephrine, were comparable to those observed in unstimulated rings (Figure 4C–E).

**FIGURE 5:**
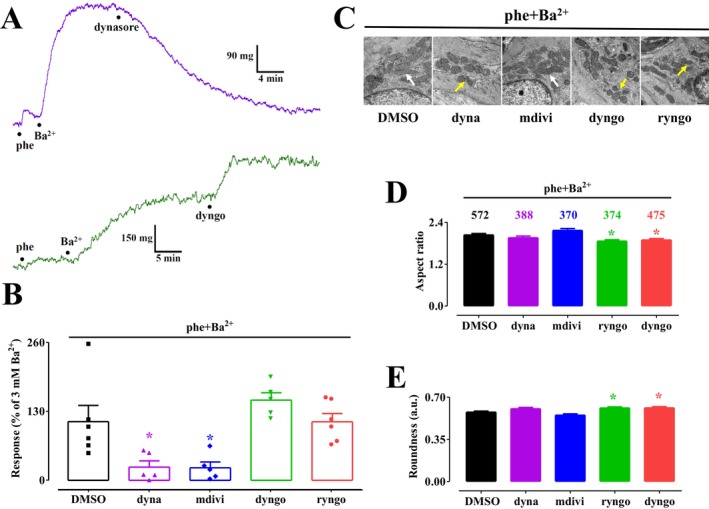
Effect of dynamin modulators on Ba^2+^‐induced contraction and mitochondrial dynamics in rat aorta rings pre‐treated with phenylephrine. (A) Traces (representative of five experiments) showing the effects of 80 μM dynasore (upper trace) and 80 μM dyngo‐4a (lower trace) on the tension developed in response to 3 mM BaCl_2_ in the presence of 1 μM phenylephrine. (B) Effect of 11.4 mM DMSO, 80 μM dynasore (dyna), 20 μM mdivi‐1 (mdivi), 80 μM dyngo‐4a (dyngo), and 50 μM ryngo 1–23 (ryngo) on ring contraction evoked by 3 mM BaCl_2_ in the presence of 1 μM phenylephrine. Columns represent the mean ± SEM (*n* = 5–6). *p < 0.05 vs. DMSO, Dunnett post hoc test. (C) Transmission electron microscope images of aorta rings contracted by 3 mM BaCl_2_ in the presence of 1 μM phenylephrine, showing the effects of 11.4 mM DMSO, 80 μM dynasore (dyna), 20 μM mdivi‐1 (mdivi), 80 μM dyngo‐4a (dyngo), and 50 μM ryngo 1–23 (ryngo) (scale bar = 500 nm). Yellow arrows point at fragmented mitochondria, whereas white arrows point at elongated mitochondria. (D, E) Summary of transmission electron microscope data: (D) aspect ratio and (E) roundness representative of mitochondrial dynamics. Columns represent the mean ± SEM, with the numbers of mitochondria analyzed per group shown above the bars. *p < 0.05 vs. DMSO, Dunnett post hoc test.

The effects of DRP1 modulators on Ba^2+^‐induced vasoconstriction in the presence of phenylephrine are illustrated in Figure 5. Notably, the structurally similar DRP1 inhibitors dynasore and dyngo‐4a exhibited contrasting effects, with dynasore strongly inhibiting and dyngo‐4a significantly enhancing Ba^2+^‐induced contraction (Figure 5A,B). Mdivi‐1 displayed a similar inhibitory effect to dynasore, while ryngo 1–23 had no significant impact (Figure 5B). Under these experimental conditions, the analysis of mitochondrial dynamics revealed that dyngo‐4a and ryngo 1–23 significantly reduced the aspect ratio and increased roundness values. In contrast, dynasore and mdivi‐1 had no observable effects on these parameters (Figure 5C–E).

### 3.3 Mechanical Activity and Mitochondrial Dynamics Assessment in (S)‐(−)‐Bay K 8644 Pre‐Treated Aorta Rings Stimulated by BaCl_2_

**FIGURE 6:**
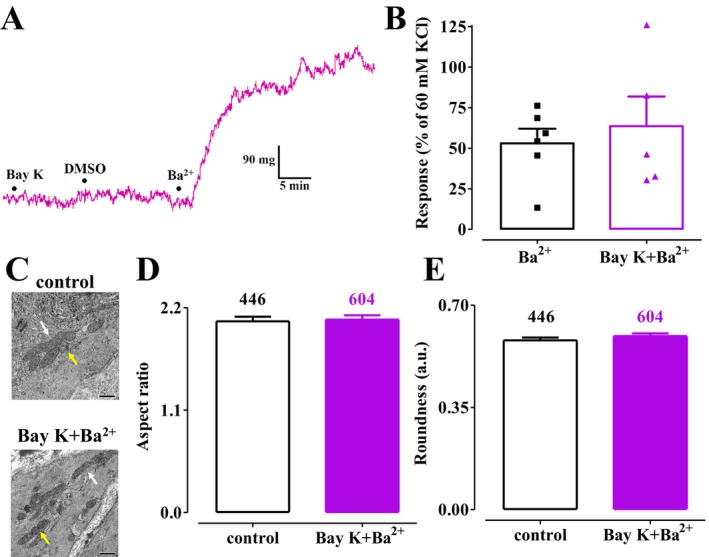
Effect of (S)‐(−)‐Bay K 8644 plus Ba^2+^ on rat aorta ring muscle tone and mitochondrial dynamics. (A) Trace (representative of five experiments) showing tension developed in response to 3 mM BaCl_2_ in the presence of 1 μM (S)‐(−)‐Bay K 8644 and the vehicle (11.4 mM DMSO). (B) Effect of 3 mM BaCl_2_, in the absence (same data as in Figure 2B) or presence of 1 μM (S)‐(−)‐Bay K 8644, on ring muscle tone. On the ordinate scale, the response is expressed as a percentage of the tension evoked by 60 mM KCl in the ring viability test. Columns represent the mean ± SEM (*n* = 5–6). (C) Transmission electron microscope images of aorta rings under control conditions and contracted by 3 mM BaCl_2_ in the presence of 1 μM (S)‐(−)‐Bay K 8644 (scale bar = 500 nm). Yellow arrows point at fragmented mitochondria, whereas white arrows point at elongated mitochondria. (D, E) Summary of transmission electron microscope data: (D) aspect ratio and (E) roundness, representative of mitochondrial dynamics. Columns represent the mean ± SEM, with the numbers of mitochondria analyzed per group shown above the bars.

This series of experiments aimed to determine whether mitochondrial fission occurred when Ba^2+^‐induced contraction was elicited in the presence of (S)‐(−)‐Bay K 8644, a Ca_V_1.2 channel stimulator [24]. As depicted in Figure 6A, (S)‐(−)‐Bay K 8644 did not alter the resting tone. The subsequent addition of Ba^2+^ produced a contractile response similar in magnitude to that of Ba^2+^ alone (Figure 6B). Mitochondrial aspect ratio and roundness values, measured after the addition of (S)‐(−)‐Bay K 8644 (1.702 ± 0.043 and 0.666 ± 0.011; *n* = 294), were not different from those measured in its absence (1.714 ± 0.047, *p* = 0.8494, and 0.672 ± 0.012, *p* = 0.7079; *n* = 295). The values measured at the plateau of the Ba^2+^‐induced contraction in the presence of (S)‐(−)‐Bay K 8644 were comparable to those observed in unstimulated rings (Figure 6C–E).

**FIGURE 7:**
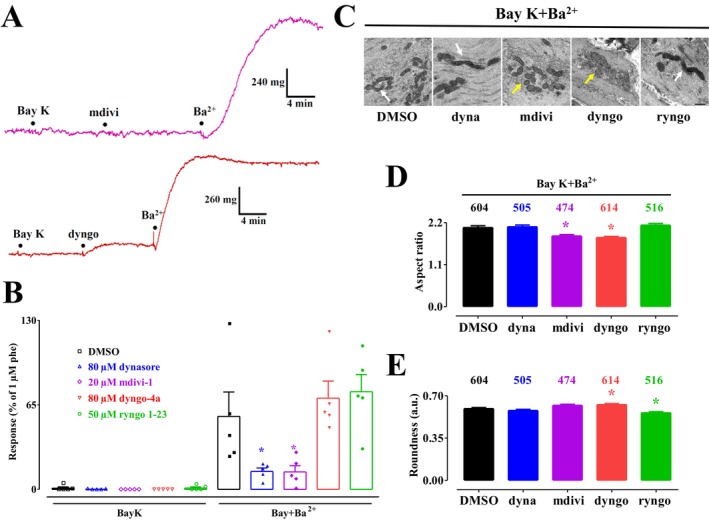
Effect of dynamin modulators on Ba^2+^‐induced contraction and mitochondrial dynamics in rat aorta rings pre‐treated with (S)‐(−)‐Bay K 8644. (A) Traces (representative of five experiments) showing the effects of 50 μM mdivi‐1 (upper trace) and 80 μM dyngo‐4a (lower trace) on the tension developed in response to 3 mM BaCl_2_ in the presence of 1 μM (S)‐(−)‐Bay K 8644. (B) Effect of 11.4 mM DMSO, 80 μM dynasore (dyna), 20 μM mdivi‐1 (mdivi), 80 μM dyngo‐4a (dyngo), and 50 μM ryngo 1–23 (ryngo) on ring muscle tone pre‐treated with 1 μM (S)‐(−)‐Bay K 8644 (left) and after the addition of 3 mM BaCl_2_ (right). Columns represent the mean ± SEM (*n* = 5). *p < 0.05 vs. DMSO, Dunnett post hoc test. (C) Transmission electron microscope images of aorta rings contracted by 3 mM BaCl_2_ in the presence of 1 μM (S)‐(−)‐Bay K 8644, showing the effect of 11.4 mM DMSO, 80 μM dynasore (dyna), 20 μM mdivi‐1 (mdivi), 80 μM dyngo‐4a (dyngo), and 50 μM ryngo 1–23 (ryngo) (scale bar = 500 nm). Yellow arrows point at fragmented mitochondria, whereas white arrows point at elongated mitochondria. (D, E) Summary of transmission electron microscope data: (D) aspect ratio and (E) roundness, representative of mitochondrial dynamics. Columns represent the mean ± SEM, with the numbers of mitochondria analyzed per group shown above the bars. *p < 0.05 vs. DMSO, Dunnett post hoc test.

The results of experiments characterizing the effects of DRP1 modulators on muscle response and mitochondrial dynamics in rings challenged by Ba^2+^ in the presence of (S)‐(−)‐Bay K 8644 are summarized in Figure 7. While the resting tone of aortic rings remained unaffected, DRP1 modulators either potentiated (dyngo‐4a +25%, ryngo 1–23 +34%) or significantly inhibited (dynasore and mdivi‐1) the response to Ba^2+^ (Figure 7B). The morphological analysis of mitochondria yielded the following results: dyngo‐4a significantly reduced the aspect ratio and increased roundness; mdivi‐1 reduced the aspect ratio without significantly altering roundness; ryngo 1–23 did not affect the aspect ratio but reduced roundness; dynasore showed no effect on either parameter (Figure 7C–E).

## 4 Discussion

Previous studies have hypothesized that activation of mitochondrial fission contributes to the development of receptor agonist‐ and membrane depolarization‐induced vascular contraction, leading to the conclusion that the two phenomena are obligatorily interconnected [17, 18]. In the present study, we demonstrate that mitochondrial fission does not occur in Ca^2+^‐depleted rat aorta rings contracted by Ba^2+^ in the absence of extracellular Ca^2+^. Similarly, mitochondrial fission was not activated when Ba^2+^‐induced contraction was evoked in the presence of either phenylephrine (an α_1_ adrenergic receptor agonist) or (S)‐(−)‐Bay K 8644 (an activator of Ca_V_1.2 channels), indicating that vascular contraction can occur without mitochondrial fission stimulation. This conclusion is further supported by the evidence that mitochondria were significantly more elongated at the plateau of Ba^2+^‐induced contraction compared to the resting vessel, suggesting inhibition, rather than activation, of mitochondrial division.

Mitochondria elongation was not observed in aortic rings contracted by Ba^2+^ in the presence of phenylephrine or (S)‐(−)‐Bay K 8644. The reasons why the replacement of Ca^2+^ with Ba^2+^ prevents the activation of mitochondrial fission during contraction can only be hypothesized. DRP1 is a cytoplasmic protein that, upon an increase in intracellular Ca^2+^, is recruited to the outer mitochondrial membrane, where it polymerizes to form a collar that induces mitochondrial division [1]. The Ca^2+^‐induced activation of DRP1 is mediated by Ca^2+^‐activated calcineurin, which dephosphorylates DRP1 at the Ser^637^ site [1]. Ca^2+^ is also essential for inner mitochondrial membrane division: it stimulates the activity of the protein INF2 [3], which promotes actin polymerization at contact sites between mitochondria and the sarcoplasmic reticulum. This process enhances Ca^2+^ efflux from the sarcoplasmic reticulum to mitochondria at these contact sites, ultimately triggering constriction and scission of the inner mitochondrial membrane [3, 4]. Based on these data, Ba^2+^ may inhibit mitochondrial fission by failing to substitute for Ca^2+^ in the activation of DRP1 and/or INF2. Alternatively, the absence of mitochondrial fission observed in our experimental model could be attributed to the depletion of the sarcoplasmic reticulum, which would preclude the INF2‐dependent fission mechanism. Lastly, the lack of mitochondrial fission during Ba^2+^‐induced contraction might be explained by the presence of Ba^2+^ within the mitochondria. Notably, Ba^2+^ is not stored in the sarcoplasmic reticulum and, therefore, cannot be released to mitochondria. However, it can translocate from the cytosol to the mitochondrial matrix, accumulating in vesicles alongside Ca^2+^ [16].

Another significant implication of the results presented here is that Ba^2+^‐induced vasoconstriction can serve as an experimental model to investigate the role of mitochondrial fission in the vascular effects of drugs and/or in the mechanisms regulating vessel tone. Mdivi‐1 remains the most extensively utilized mitochondrial fission inhibitor, with over 700 publications since its discovery in 2008 [25]. It acts by inhibiting DRP1 activity and polymerization, thereby preventing the formation of the mitochondrial division collar [26]. The use of mdivi‐1 has significantly advanced our understanding of the role of mitochondrial fission in both physiological and pathological processes, highlighting this phenomenon as a potential therapeutic target [27, 28]. Previous studies on in vitro vascular smooth muscle preparations demonstrated that contractions induced by phenylephrine, endothelin‐1, and KCl are associated with mitochondrial fission and increased reactive oxygen species (ROS) formation. As all these effects were inhibited by mdivi‐1, the authors proposed that mitochondrial fission activation plays a key role in the development of vascular contraction via ROS production [17, 18]. However, recent studies have revealed that mdivi‐1 exerts its vasorelaxant effects independently of DRP1 inhibition. Specifically, at concentrations that induce vasodilation, mdivi‐1 blocks sarcolemmal Ca_V_1.2 channels and stimulates K_Ca_1.1 channels, reducing intracellular Ca^2+^ levels. This dual action accounts for inhibiting mitochondrial fission and the ability to relax vascular smooth muscle [19, 20]. Furthermore, mdivi‐1 reduces Ca^2+^ concentration in neuronal cells [29] and ROS formation in fibroblasts, COS‐7 cells, and DRP1 knockout cells, without affecting mitochondrial dynamics. These findings suggest that mdivi‐1 effects are independent of DRP1 inhibition [30].

In the present study, mdivi‐1 caused a near‐complete reversal of Ba^2+^‐induced contractions, in the absence and presence of phenylephrine and (S)‐(−)‐Bay K 8644. Since the contraction in this experimental model was not accompanied by mitochondrial fission, it can be hypothesized that the vasorelaxant activity of mdivi‐1 is mediated through mechanisms unrelated to DRP1 inhibition. This hypothesis is further supported by the observation that the mitochondrial shape in aorta rings at the plateau of contraction induced by various stimuli was similar to that observed in preparations relaxed by mdivi‐1. This suggests a dissociation between the spasmolytic effect and the inhibition of mitochondrial fission.

**FIGURE 8:**
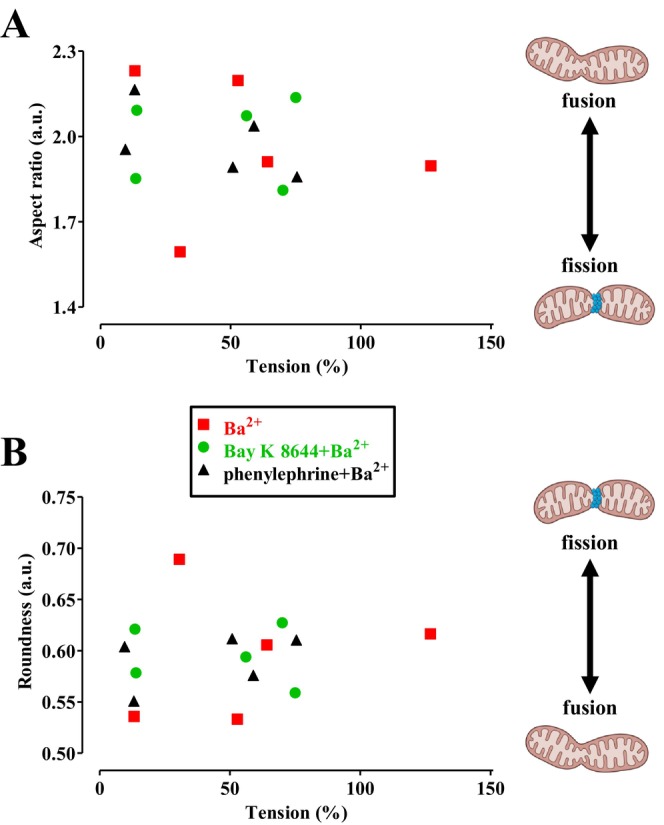
Linear correlation analysis of vascular smooth muscle tension and mitochondrial dynamics. (A, B) Data points represent tension (expressed as a percentage of control) and (A) aspect ratio or (B) roundness pairs obtained under four different conditions in the presence of dynamin modulators or vehicle alone. (A) Pearson correlation coefficient: −0.1898, *p* = 0.4981. (B) Pearson correlation coefficient: 0.1518, *p* = 0.5892.

Interestingly, dynasore, another DRP1 inhibitor primarily used to inhibit cellular endocytosis [31], inhibits vascular Ca_V_1.2 channels and stimulates K_Ca_1.1 channels in a DRP‐1‐independent manner [20]. In the present study, dynasore exhibited a behavior comparable to mdivi‐1. This evidence supports the hypothesis that inhibition of mitochondrial fission is not necessarily correlated with the vasorelaxant activity of DRP1 inhibitors. This conclusion is further reinforced by the observation that dyngo‐4a, a structural analogue of dynasore that is 10 times more potent as a DRP1 inhibitor [32], enhanced Ba^2+^‐induced contraction and altered mitochondrial shape, reducing their length and increasing their roundness—effects that suggest a stimulatory rather than inhibitory action on mitochondrial fission. Notably, similar results were observed with ryngo 1–23, an activator of DRP1 and mitochondrial fission [33]. Finally, a linear correlation between muscle tension and either aspect ratio or roundness, recorded in the presence of vehicle or DRP1 modulators, was examined. As shown in Figure 8, no such correlation was found.

## 5 Conclusion

In conclusion, the present results and previous studies [19, 20] establish that mitochondrial fission is not a critical step for smooth muscle contraction in the aorta. Furthermore, DRP1 modulators, commonly used to investigate the role of mitochondrial fission in physiological processes and pharmacological studies, exhibit off‐target effects that can lead to misinterpretation of experimental outcomes. It remains to be determined why mitochondrial fission occurs in vessels stimulated in the presence of Ca^2+^, and whether this is merely a “secondary effect” or plays a different role. Using Ba^2+^ as a vasoconstricting agent provides a valuable model to further investigate the dynamin dependence of biological processes regulating vascular tone and evaluate the potential off‐target effects of dynamin inhibitors.

## Author Contributions

**Mariangela Gentile:** investigation; validation; visualization; writing – original draft. **Alice Panti:** investigation; validation; visualization. **Eugenio Paccagnini:** investigation; validation; visualization; writing – original draft. **Pietro Lupetti:** supervision; review and editing. **Sergio Bova:** supervision; writing – review and editing. **Fabio Fusi:** conceptualization; investigation; supervision; validation; visualization; writing – original draft; writing – review and editing.

## Conflicts of Interest

The authors declare no conflicts of interest.

## Data Availability

The data that support the findings of this study are available from the corresponding author, F.F., upon reasonable request.
